# Virus-specific Dicer-substrate siRNA swarms inhibit SARS-CoV-2 infection in TMPRSS2-expressing Vero E6 cells

**DOI:** 10.3389/fmicb.2024.1432349

**Published:** 2024-11-14

**Authors:** Miao Jiang, Larissa Laine, Pekka Kolehmainen, Laura Kakkola, Veera Avelin, Elina Väisänen, Minna M. Poranen, Pamela Österlund, Ilkka Julkunen

**Affiliations:** ^1^Microbiology Unit, Finnish Institute for Health and Welfare, Helsinki, Finland; ^2^Infection and Immunity, Institute of Biomedicine, University of Turku, Turku, Finland; ^3^Molecular and Integrative Biosciences Research Programme, Faculty of Biological and Environmental Sciences, University of Helsinki, Helsinki, Finland; ^4^Clinical Microbiology Unit, Turku University Central Hospital, Turku, Finland

**Keywords:** COVID-19, SARS-CoV-2, viral replication, gene silencing, RNA interference, siRNA swarm, Dicer-substrate siRNA (DsiRNA)

## Abstract

After 4 years of the COVID-19 pandemic, SARS-CoV-2 continues to circulate with epidemic waves caused by evolving new variants. Although the rapid development of vaccines and approved antiviral drugs has reduced virus transmission and mitigated the symptoms of infection, the continuous emergence of new variants and the lack of simple-use (non-hospitalized, easy timing, local delivery, direct acting, and host-targeting) treatment modalities have limited the effectiveness of COVID-19 vaccines and drugs. Therefore, novel therapeutic approaches against SARS-CoV-2 infection are still urgently needed. As a positive-sense single-stranded RNA virus, SARS-CoV-2 is highly susceptible to RNA interference (RNAi). Accordingly, small interfering (si)RNAs targeting different regions of SARS-CoV-2 genome can effectively block the expression and replication of the virus. However, the rapid emergence of new SARS-CoV-2 variants with different genomic mutations has led to the problem of viral escape from the targets of RNAi strategy, which has increased the potential of off-target effects by siRNA and decreased the efficacy of long-term use of siRNA treatment. In our study, we enzymatically generated a set of Dicer-substrate (D)siRNA swarms containing DsiRNAs targeting single or multiple conserved sequences of SARS-CoV-2 genome by using *in vitro* transcription, replication and Dicer digestion system. Pre-transfection of these DsiRNA swarms into Vero E6-TMPRSS2 cells inhibited the replication of several SARS-CoV-2 variants, including the recent Omicron subvariants BQ.1.1 and XBB.1.5. This *in vitro* investigation of novel DsiRNA swarms provides solid evidence for the feasibility of this new RNAi strategy in the prevention and treatment of SARS-CoV-2 infection.

## 1 Introduction

Since the outbreak of the coronavirus disease 2019 (COVID-19) in Wuhan, China, at the end of 2019, people have suffered from a devastating pandemic for over 4 years with more than 775 million infected and 7 million deaths globally. According to the World Health Organization (WHO) COVID-19 is no more considered as a Public Health Emergency of International Concern (announcement on 5^th^ of May 2023). However, the severe acute respiratory syndrome coronavirus 2 (SARS-CoV-2), the causative agent of COVID-19, continues to circulate with epidemic waves all over the world and new viral variants are still emerging.

In order to prevent and reduce the severity and mortality of the pandemic, vaccines and antiviral drugs against COVID-19 were rapidly developed and used as repurposed drugs, respectively. Several types of COVID-19 vaccines have been authorized and widely used, including traditional protein-based vaccine (Suryawanshi, 2023), adenoviral vector vaccine with the most advanced technology (Mendonça et al., 2021), and novel mRNA vaccines (Gote et al., 2023). Therapeutic drugs against COVID-19 have also been intensively and systematically studied, targeting either viral or host proteins essential for viral replication in order to block the viral life cycle and to evoke or enhance broad-spectrum antiviral activities (Mousavi et al., 2022; Li et al., 2023).

RNA interference (RNAi) is a conserved gene regulation mechanism in eukaryotic cells that is triggered by small interfering RNAs (siRNAs) (Dana et al., 2017). SiRNAs mediate the recognition of complementary mRNAs in the cytoplasm (Hammond et al., 2001) or target and remodel chromatin in the nucleus (Morris, 2008), leading to the degradation of target mRNAs as post-transcriptional gene silencing or heterochromatinization of the targeted gene as transcriptional gene silencing, respectively (Dudley and Goldstein, 2003). RNAi has been applied as a novel RNA therapeutic technology in the treatment of many diseases, including cancer (Tian et al., 2021), inherited disorders (Seyhan, 2011), and viral infections (Ge et al., 2003; Tompkins et al., 2004; Li et al., 2005; Wilson and Richardson, 2006; DeVincenzo et al., 2010).

SARS-CoV-2 is highly susceptible to RNAi since its positive-sense single-stranded RNA genome, subgenomic RNAs, and intermediate viral RNAs (vRNAs) generated during the virus replication can be targeted by siRNAs and the RNAi machinery. Accordingly, several studies have demonstrated the antiviral therapeutic potential of siRNAs targeting different SARS-CoV-2 genes and essential noncoding regions, including 5′- and 3′-untranslated regions (UTR) (Idris et al., 2021; Tolksdorf et al., 2021; Ambike et al., 2022), ORF1 (Ambike et al., 2022), genes encoding the nonstructural protein (NSP) 7, NSP 8 and NSP 12 forming the viral replication machinery (Idris et al., 2021; Khaitov et al., 2021; Shawan et al., 2021), NSP 13 helicase gene (Idris et al., 2021), and genes encoding the structural proteins S, M, and N (Wu and Luo, 2021; Ambike et al., 2022). All these studies have been based on the use of chemically synthesized traditional siRNAs. Furthermore, siRNAs targeting additional SARS-CoV-2 genes have been designed and their therapeutic potential has been predicted, but the antiviral efficacy of such siRNAs needs to be further experimentally validated (Ghosh et al., 2020; Saadat, 2022; Nawaz et al., 2023).

SiRNA-based treatments have several advantages over other antiviral drug therapies. SiRNAs can be quickly designed and produced at a relatively low cost, a wide range of siRNA targets can be easily identified in viral genomes, and the efficacy of siRNA treatment is typically high. However, the rapid mutation rate of SARS-CoV-2 genome may increase the potential emergence of viral escape variants which no longer share sequence identity with the antiviral siRNA. This phenomenon may increase the potential of off-target effects of siRNA and decrease the efficacy of long-term use of siRNA treatment. As an alternative for the traditional single-site siRNAs, we and others have introduced the concept of antiviral siRNA swarms (Romanovskaya et al., 2012; Paavilainen et al., 2017; Jiang et al., 2019; Kalke et al., 2020). The siRNA swarms contain tens or hundreds of target-specific siRNA molecules to minimize viral escape and to counter the genetic diversity in viral populations, as successfully shown in our previous studies on influenza A virus (Jiang et al., 2019) and herpes simplex virus (Romanovskaya et al., 2012; Paavilainen et al., 2017; Kalke et al., 2020). Moreover, the low concentration of each individual siRNA in the swarm likely reduces the risk of severe off-target effects (Jiang et al., 2019; Levanova et al., 2020; Levanova and Poranen, 2024). Instead of the canonical 21-mer siRNAs we utilize 25-27-mer Dicer-substrate siRNAs (DsiRNAs) which upon introduction into a cell are processed by the cellular Dicer, resulting in enhanced RNAi potency and efficacy (Kim et al., 2005). Previous studies have demonstrated the potent antiviral efficacy of DsiRNA swarms against herpes simplex virus as well as low cytotoxicity of the treatment *in vitro* and *in vivo* (Romanovskaya et al., 2012; Paavilainen et al., 2015, 2017; Kalke et al., 2022; Lasanen et al., 2023). In the present study, we enzymatically generated a set of DsiRNA swarms, each targeting single or multiple conserved regions in the SARS-CoV-2 genome. We screened the antiviral efficacy of the produced DsiRNA swarms targeting 5′-UTR leader sequence, 3′-UTR, 15 NSPs and four structural proteins against SARS-CoV-2 infection in TMPRSS2-expressing Vero E6 (VE6-T2) cells. Furthermore, we investigated the antiviral efficacy of selected DsiRNA swarms against the infection of different SARS-CoV-2 variants, including recent Omicron sub-variants. Our investigation provides solid evidence for the feasibility of several SARS-CoV-2 specific siRNA swarms for the prophylaxis and treatment of SARS-CoV-2 infection.

## 2 Materials and methods

### 2.1 Cell cultures

Cultured Vero E6 cells constitutively expressing type II transmembrane serine protease (Vero E6-TMPRSS2-H10, VE6-T2) (Rusanen et al., 2021) were maintained by continuous growth in Eagle minimal essential medium (Eagle-MEM) (Sigma-Aldrich) supplemented with 0.6 μg/ml penicillin, 60 μg/ml streptomycin, 2 mM l-glutamine, 20 mM HEPES, and 10% (vol/vol) fetal bovine serum (Integro). Cells were maintained at 37°C in a humidified atmosphere in the presence of 5% CO_2_.

### 2.2 Design of the chimeric and gene-specific DsiRNA swarms against SARS-CoV-2

Complete sequences of 15 NSP genes (excluding NSP11 peptide due to its small, limited size), 4 structural protein genes, as well as 5′ and 3′-UTR of SARS-CoV-2 Wuhan-Hu-1 strain (Accession NC_045512, Version NC_045512.2) were synthesized and cloned into pEBB-N-HA vector by GeneArt (Thermo Fisher Scientific). Similarly, a chimeric gene construct combining seven 400 bp-long parts of SARS-CoV-2 genome (total length 2,800 bp) was synthesized by GeneArt and cloned into pMK-RQ vector (GeneArt). The produced chimeric construct contains sequences derived from ORF1ab and M and N genes, including sequences 7600–7999, 10571–10970, 12094–12493, 14819–15218, 16928–17327, 26816–27215, and 28665–29664 (numbering according to Wuhan-Hu-1).

### 2.3 DsiRNA preparations

SARS-CoV-2 sequences were PCR amplified from the plasmids harboring viral sequences (described in 2.2.) and the PCR products were used as templates for dsRNA synthesis. DsiRNAs were generated using bacteriophage T7 DNA-dependent RNA-polymerase and bacteriophage ϕ6 RNA-dependent RNA-polymerases (Aalto et al., 2007). Non-specific control dsRNA was produced similarly from the *Escherichia coli lacI* gene in plasmid pET32b (Levanova et al., 2020). DsiRNA swarms were subsequently generated from the produced long dsRNAs using recombinant *Giardia intestinalis* Dicer (Paavilainen et al., 2017). All produced siRNAs were desalted in NAP5 columns (GE Healthcare) as described previously (Romanovskaya et al., 2013).

### 2.4 SARS-CoV-2 viruses

The variants of SARS-CoV-2 used in the study were an early strain Fin-3 (B.1.1.29, hCoV-19/Finland/FIN-3/2020, GISAID EPI_ISL_2365908, GenBank ON531991), Omicrons BQ.1.1 (hCoV-19/Finland/THL-202219039/2022, EPI_ISL_15762173, OQ411064), and XBB.1.5 (hCoV-19/Finland/THL-22430/2022, EPI_ISL_16526646, OQ509907). The viruses were isolated from the nasopharyngeal swabs obtained from COVID-19 patients and the virus sequences were obtained by whole genome sequencing using Illumina Miseq (Jalkanen et al., 2021). Fin-3 and BQ.1.1 viruses were isolated in VE6-T2 cells with two passages. Virus stocks were collected 3 days after plating of the second passage. For the XBB.1.5 virus, a third passage was required to increase virus yield and the stock was collected after 3 days. Virus titers (TCID_50_/ml) were determined with an endpoint dilution assay in VE6-T2 cells: Fin-3 titer was 1 × 10^8^ TCID_50_/ml, BQ.1.1 was 1 × 10^6^ TCID_50_/ml, and XBB.1.5 was 1 × 10^7^ TCID_50_/ml. All experiments using infective SARS-CoV-2 virus strains were performed within a BSL-3 laboratory of the Finnish Institute for Health and Welfare, Helsinki, Finland.

### 2.5 DsiRNA transfection and SARS-CoV-2 infection in VE6-T2 cells

VE6-T2 cells were plated onto 12-well culture plates (5 × 10^5^ cells/well) and after 1 day transfected with different DsiRNA swarms (20 nM) using TransIT-X2 transfection reagent (TransIT-X2 Dynamic Delivery System; Mirus Bio) according to the manufacturer's instructions. At 21 h post transfection (p.t.), the cells were infected with different SARS-CoV-2 variants for 24 h or 48 h. The supernatants and cells were collected, and used for TCID_50_ assay, isolation of supernatant vRNA and total cellular RNA, or lysed in passive lysis buffer (Dual-Luciferase Reporter Assay System; Promega) for the subsequent analyses.

### 2.6 Quantitative reverse transcriptase PCR

Total cellular RNA was isolated from VE6-T2 cells using RNeasy Mini kit (Qiagen). DNase-treated total cellular RNA was reverse transcribed into complementary (c)DNA by using the TaqMan Reverse Transcriptase kit (Applied Biosystems). cDNA samples were then amplified using TaqMan Universal PCR Master Mix (Applied Biosystems) and a commercial gene expression system assay (Applied Biosystems) with primers and probes for human interferon (IFN) gene, *IFN-*λ*1* (Hs00601677_g1, 93% identical to green monkey *IFN-*λ*1* gene). A SARS-CoV-2 E gene-specific primer-probe pair was used to detect vRNAs of all SARS-CoV-2 viruses (Corman et al., 2020). Each cDNA sample was amplified in duplicate with an Mx3005P quantitative PCR system (Stratagene). The relative amount of cytokine or viral RNAs was calculated with the delta-delta comparative threshold cycle (ΔΔCT) method using human18S rRNA levels (Ribosomal RNA Control Reagents VIC™ Probe, 99% identical to green monkey *18S* gene, Applied Biosystems) in the standardization, and comparing the expression levels in relation to the untreated mock samples.

To quantify viral RNA from the supernatant samples, non-transfected and DsiRNA-transfected VE6-T2 cells were infected as described above, and the supernatant samples were collected at 1 h, 24 h and 48 h post infection (p.i.). RNA isolation and cDNA synthesis have been described previously (Jiang et al., 2021). RT-qPCR was performed using a Qiagen QuantiTect Multiplex PCR NoRox kit (Qiagen) with the same SARS-CoV-2 E gene-specific primer-probe pair as described above.

### 2.7 Western blot analysis

Whole-cell lysates were prepared from VE6-T2 cells with passive lysis buffer (Promega). Protein aliquots of whole-cell lysates (30 μg) were separated on 10% SDS-polyacrylamide gels using a Laemmli buffer system (Laemmli, 1970). Proteins were transferred onto Immobilon-P membranes, followed by blocking with 5% milk in PBS. Previously described antibodies against SARS-CoV-2 S and SARS-CoV N proteins were used (Jiang et al., 2021). As a loading control, glyceraldehyde-3-phosphate dehydrogenase (GAPDH) was detected with anti-GAPDH (Cell Signaling Technology). Horseradish peroxidase (HRP)-conjugated goat anti-rabbit antibody (DakoCytomation) was used as the secondary antibody. Antibody binding was visualized by an enhanced chemiluminescence (ECL) system (Pierce ECL Western Blotting substrate; Thermo Fisher Scientific) in iBright Imaging System (Thermo Fisher Scientific).

### 2.8 Endpoint dilution assay

Vero E6-T2 cells were cultured and plated into 96-well plates 24 h prior to infection. Serial dilutions of each supernatant collected from SARS-CoV-2 infected cells at different time points were made and eight parallel wells were inoculated with each sample dilution. Cytopathic effect (CPE) was observed under light microscope at day 5 p.i. and each well was scored either positive or negative for virus infection. The Spearman-Kärber method was used to calculate the results, presented as log TCID_50_/ml.

### 2.9 Statistical analyses

Results of experiments are presented as means ± the standard error of mean (SEM) of the means. Statistical analyses were performed using unpaired, two-tailed Student's *t*-test. The differences were considered to be statistically significant when *P* < 0.05.

### 2.10 Cytotoxicity assay

In order to determine the cytotoxicity induced by DsiRNAs and the infection of SARS-CoV-2, the amount of lactate dehydrogenase (LDH) released by lysed cells into the culture medium, was measured from the supernatant of the samples. CytoTox96 Non-Radioactive Cytotoxicity Assay kit (Promega, USA) was used to measure the level of LDH via a coupled enzymatic assay. The level of LDH is proportional to the final product, red formazan, which was measured at 492 nm with a Multiscan™ FC Microplate Photometer (Thermo Fisher Scientific). The average absorbance values of the culture medium background from non-infected or mock samples were subtracted from the sample values, and the percentage of cytotoxicity was calculated with the following formula:

Percent cytotoxicity = 100 × Experimental LDH Release (OD492)/Maximum LDH Release (OD492).

## 3 Results

### 3.1 Design of SARS-CoV-2 cDNA constructs and DsiRNA swarm production

**Figure 1:**
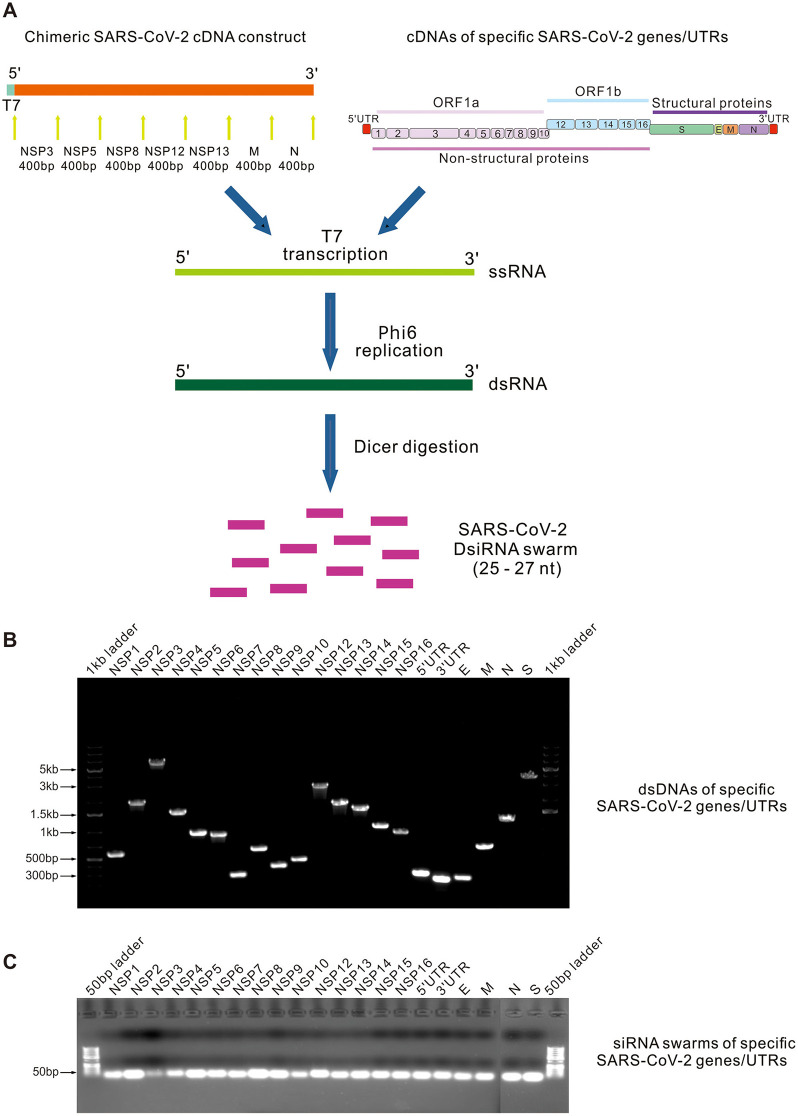
Enzymatic synthesis of SARS-CoV-2-specific DsiRNA swarms. **(A)** Schematic representation of enzymatic synthesis of DsiRNA swarms. Chimeric SARS-CoV-2 cDNA construct containing 400-bp-long conserved sequences derived from seven SARS-CoV-2 proteins coding regions (top left orange bar), and the complete sequences of 15 non-structural protein (NSP) genes, 4 structural protein genes and 5′/3′-UTR (top right bars) of SARS-CoV-2 Wuhan-Hu-1 strain were PCR amplified to produce dsDNA templates for RNA synthesis. SsRNA molecules were first transcribed using bacteriophage T7 RNA polymerase and subsequently used as templates for the bacteriophage ϕ6 RNA-dependent RNA polymerase to produce corresponding dsRNA molecules. Produced dsRNA molecules were digested into a swarm of DsiRNA molecules (SARS-CoV-2 DsiRNA swarm) using a recombinant *Giardia intestinalis* Dicer. **(B, C)** Native agarose gel electrophoresis of the PCR amplified SARS-CoV-2 gene/UTR -specific dsDNAs **(B)** and the corresponding purified DsiRNA swarms **(C)**.

Twenty-one cDNA constructs representing different SARS-CoV-2 genes or genomic noncoding regions, including 15 non-structural protein (NSP) genes (excluding NSP11 due to its limited size hampering enzymatic DsiRNA swarm production), 4 structural protein genes (S, E, M, and N), as well as 5′- and 3′-UTR were designed to screen the most potent RNAi targets in SARS-CoV-2 for DsiRNA swarm treatment (Figure 1A). In addition, a 2,800 bp-long chimeric SARS-CoV-2 construct comprising seven 400-bp-long sequences from different protein-coding regions of SARS-CoV-2 genome was designed. The sequences for the chimera were selected based on their conservation in comparison to SARS HKU-39849 (Accession no: AY278491.2).

Full-length dsRNA molecules corresponding to the designed SARS-CoV-2-specific cDNA constructs were produced *in vitro* using bacteriophage T7 DNA-dependent RNA polymerase and bacteriophage ϕ6 RNA-dependent RNA polymerase (Figure 1B), followed by digestion of the produced long dsRNAs with *Giardia intestinalis* Dicer to obtain swarms of 25 - to 27-nt-long SARS-CoV-2 DsiRNAs (Figure 1C) (Jiang et al., 2019). PET-DsiRNA swarm, derived from the *E. coli lacI* gene, was generated in a similar fashion.

### 3.2 Screening of chimeric and single gene SARS-CoV-2 DsiRNA swarms for their inhibitory efficiency against the replication of SARS-CoV-2 in VE6-T2 cells

**Figure 2:**
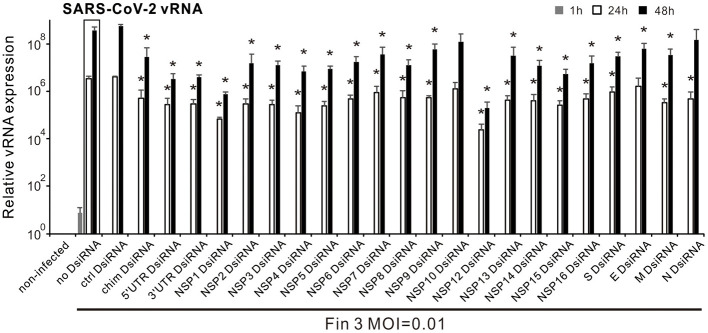
Inhibitory effect of DsiRNA swarms against SARS-CoV-2 infection in VE6-T2 cells. Cells were mock transfected (non-infected and no-DsiRNA controls) or pre-transfected with control (ctrl) DsiRNA, the indicated SARS-CoV-2 gene/UTR-specific DsiRNAs, or the chimeric (chim) DsiRNA swarm for 21 h and then infected with Fin-3 SARS-CoV-2 at a MOI of 0.01. Cells were collected at 1, 24, and 48 h p.i., and SARS-CoV-2 vRNA expression was analyzed from isolated total cellular RNA samples by RT-qPCR. The SARS-CoV-2 E vRNA Ct values were normalized against human 18S rRNA, and the relative vRNA levels were calculated by the ΔΔCT method using untreated, non-infected culture for calibration. The means (±SEM) of three parallel analyses are shown. Data is a representative of three individual experiments. Statistical significance was determined against results of samples of DsiRNA non-transfected cells (boxed bars). *P < 0.05.

To study the antiviral effect of the produced SARS-CoV-2 DsiRNA swarms, different DsiRNAs as well as the control PET-DsiRNA were transfected into VE6-T2 cells, followed by an infection with an early ancestral virus strain Fin-3 of SARS-CoV-2 at 21 h p.t. at multiplicity of infection (MOI) of 0.01. The optimal transfection amount of DsiRNA for the inhibition of SARS-CoV-2 replication was evaluated by a dose-dependent assay in VE6-T2 cells (Supplementary Figure S1). No significant changes in cell viability were observed after DsiRNA transfection (Supplementary Figure S3). Total RNAs were collected from Fin-3 infected VE6-T2 cells at 1, 24, and 48 h p.i., and SARS-CoV-2 vRNA expression was analyzed by gene-specific RT-qPCR. Compared with the 1 h incoming virus sample, the vRNA expression levels with or without pre-transfection of DsiRNAs increased at 24 h p.i. and reached a peak at 48 h p.i. (Figure 2). Pre-transfection with the control DsiRNA did not influence vRNA expression in VE6-T2 cells and the measured vRNA levels were comparable to the sample without pre-transfection of any DsiRNA. Instead, almost all SARS-CoV-2 single gene/UTR -specific DsiRNA swarms as well as the chimeric DsiRNA swarm inhibited the replication of Fin-3 to varying degrees (Figure 2). Among all the DsiRNA swarms, 5′UTR, 3′UTR, NSP1, and NSP12 DsiRNA swarms had the strongest inhibitory effect against SARS-CoV-2 infection with 1-log reduction of vRNA expression in infected cells at 24 h and over 2-log reduction at 48 h p.i.. In contrast, NSP10 and N DsiRNA swarms failed to efficiently inhibit the replication of Fin-3 (Figure 2).

### 3.3 Inhibition of Fin-3 replication by chimeric, 3′UTR, NSP1, NSP2, and NSP12 DsiRNA swarms in VE6-T2 cells

**Figure 3:**
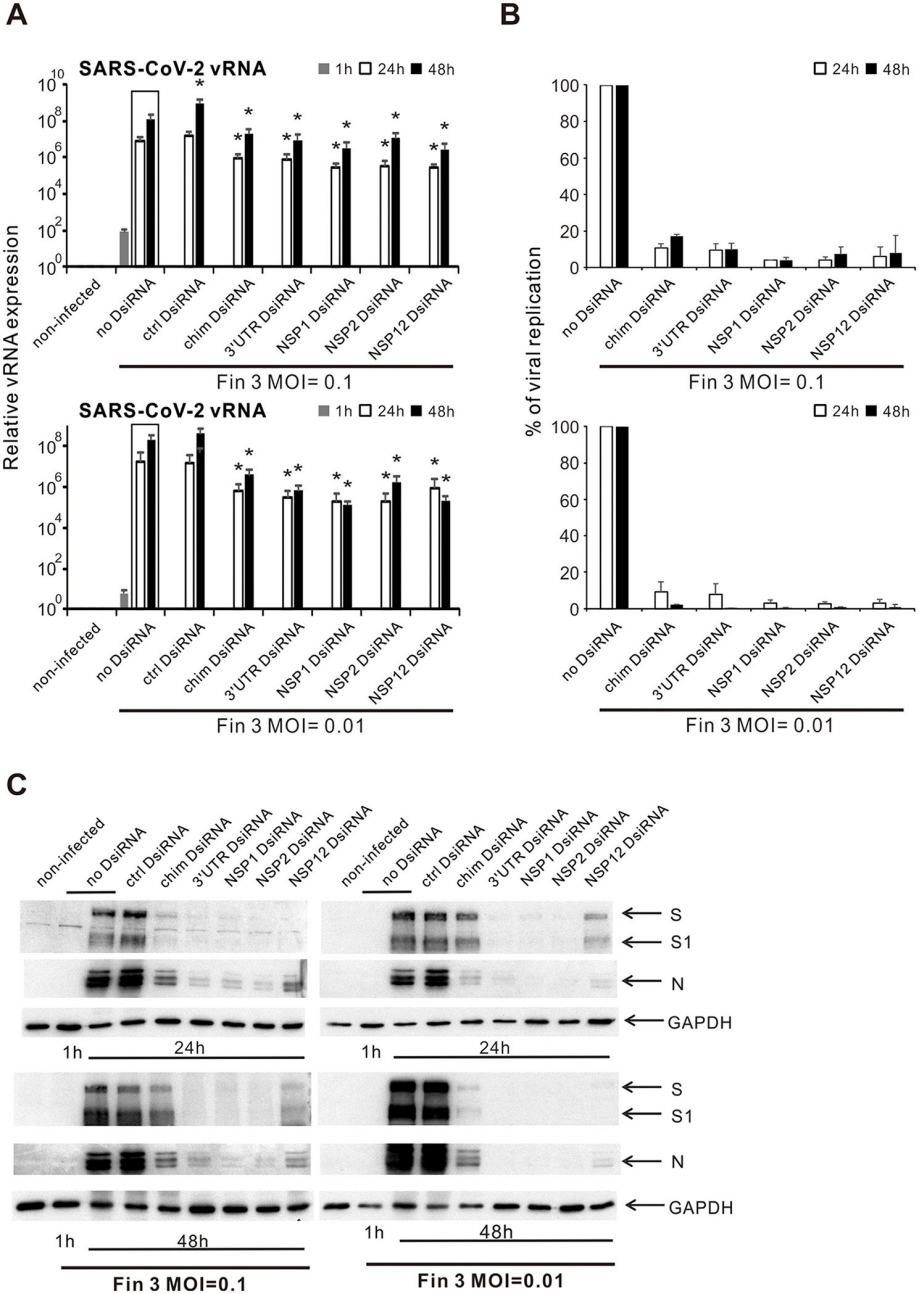
Inhibition of SARS-CoV-2 replication in VE6-T2 cells by pre-transfection with selected SARS-CoV-2 DsiRNA swarms. Cells were mock transfected (non-infected and no-DsiRNA controls) or pre-transfected with control (ctrl) DsiRNA, chimeric (chim) DsiRNA or selected SARS-CoV-2 specific DsiRNAs for 21 h. Cells were subsequently infected with Fin-3 SARS-CoV-2 at a MOI of 0.1 or 0.01. **(A)** Cells were collected at 1, 24 and 48 h p.i., and SARS-CoV-2 vRNA expression was analyzed from isolated total cellular RNA samples by RT-qPCR. The SARS-CoV-2 E vRNA Ct values were normalized against human 18S rRNA, and relative SARS-CoV-2 vRNA levels were calculated by the ΔΔCT method using untreated cellular RNA as a calibrator. The means (±SEM) of three parallel analyses are shown. Data is a representative of three individual experiments. Statistical significance was determined against results of samples of DsiRNA non-transfected cells (boxed bars). *P < 0.05. **(B)** To show the inhibition percentage of viral replication, SARS-CoV-2 vRNA expression level in DsiRNA-transfected cells were compared to non-transfected cells (as 100%, no DsiRNA), and the percentage ratio of viral replication was shown. **(C)** Western blot analysis of the expression of viral S and N proteins and GAPDH protein in DsiRNA-transfected VE6-T2 cells. Cells were collected at 24 h or 48 h post infection, and whole-cell lysates were prepared. Cellular proteins (30 μg/lane) were separated by 10% SDS-PAGE, followed by electrophoretic transfer of the proteins onto polyvinylidene difluoride membranes and visualization of the transferred proteins by protein-specific antibodies, as indicated. The data of one representative experiment of three independent experiments is shown.

Next, we chose the chimeric and four gene/UTR-specific DsiRNA swarms (3′UTR, NSP1, NSP2, and NSP12) to further analyze their inhibitory efficacy against the replication of Fin-3 in VE6-T2 cells. Chosen DsiRNA swarms were pre-transfected into VE6-T2 cells, followed by an infection with Fin-3 at 21 h p.t. at MOI 0.1 and MOI 0.01. Subsequently, total RNAs were isolated from the infected cells at 1, 24, and 48 h p.i., and vRNA expression of the SARS-CoV-2 was analyzed by gene-specific RT-qPCR. Regardless of the MOI (0.1 or 0.01), the vRNA expression levels in Fin-3 infected cells, with and without DsiRNA transfection, increased at 24 h and 48 h p.i. as compared with the 1 h input virus sample (Figure 3A). Pre-transfection of cells with the control DsiRNA swarm did not inhibit vRNA expression in Fin-3 infected cells at both MOI 0.1 and MOI 0.01, whereas pre-transfection of the chimeric and the four chosen gene/UTR-specific DsiRNA swarms led to ca. 1-log significant reduction in the expression level of vRNA at 24 h p.i. and up to 2-log significant reduction at 48 h p.i. compared with the non-transfected Fin-3 infected control (Figure 3A). Overall, the inhibitory efficacy of DsiRNA swarms against the replication of Fin-3 was better in Fin-3 infected VE6-T2 cells at a low MOI value of 0.01 as compared to the higher MOI value of 0.1 (Figure 3B).

The antiviral effects of SARS-CoV-2 DsiRNAs on viral protein expression were analyzed by Western blotting using protein samples from Fin-3 infected cells. The infection of Fin-3 at both MOI 0.1 and MOI 0.01 without any DsiRNA pre-transfection led to a very strong S and N protein expression at both 24 h p.i. and 48 h p.i. (Figure 3C). S1 subunit cleavage was more apparent at 48 h p.i. compared to 24 h p.i. in Fin-3 infected cells without DsiRNA pre-transfection at both MOI values. The pre-transfection of control DsiRNAs showed no antiviral effect against the infection of Fin-3 at both MOI 0.1 and MOI 0.01. However, the expression of viral S and N proteins and the cleavage of S1 were clearly reduced if the cells were pre-transfected with chimeric or NSP12-specific SARS-CoV-2 DsiRNAs, and almost completely inhibited by the pre-transfection of 3′UTR-, NSP1-, and NSP2-specific DsiRNAs (Figure 3C).

### 3.4 Inhibition of the productive Fin-3 SARS-CoV-2 infection in VE6-T2 cells by the chimeric, 3′UTR, NSP1, NSP2, and NSP12 DsiRNA swarms

**Figure 4:**
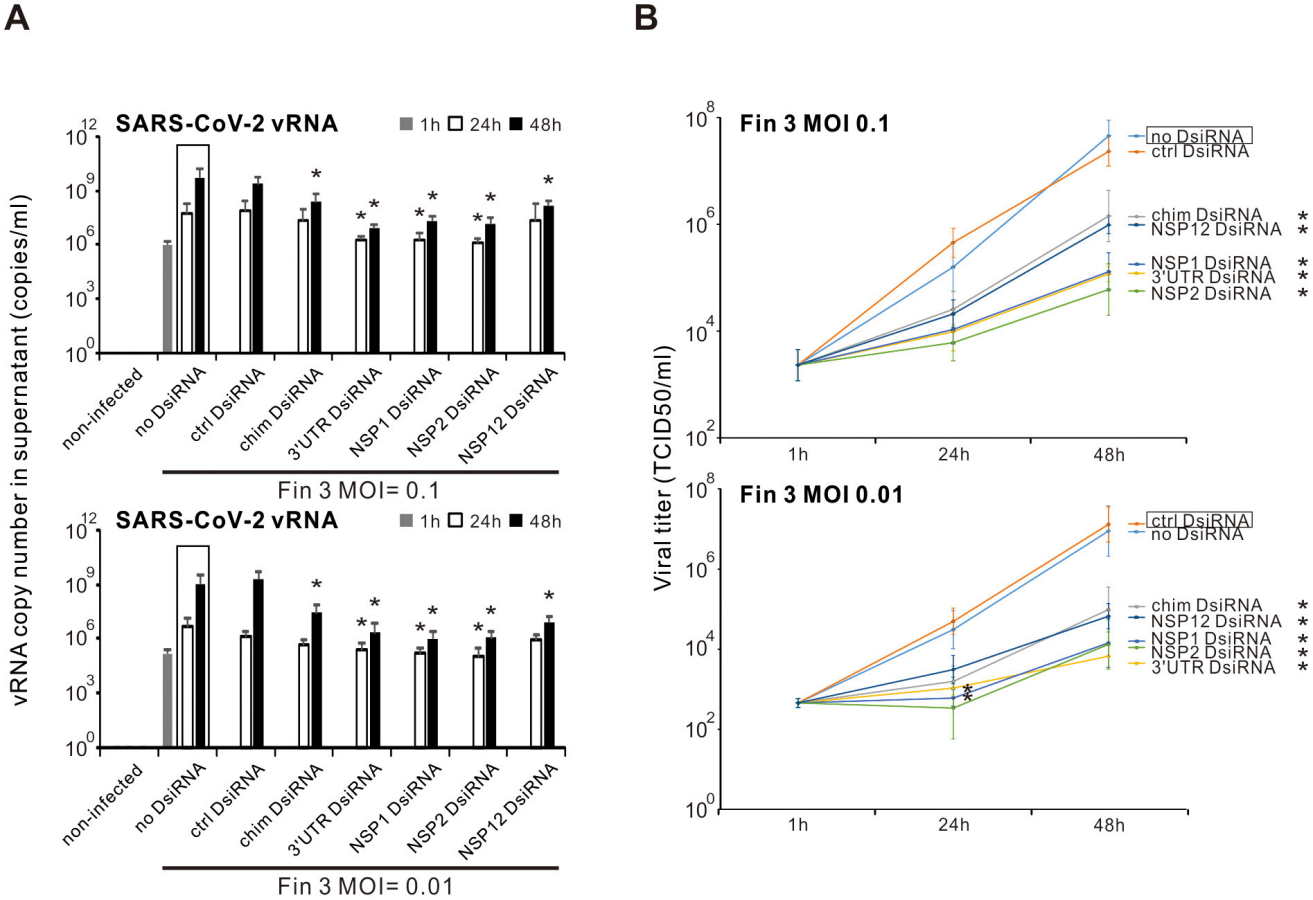
Inhibition of the productive SARS-CoV-2 infection by pre-transfection of VE6-T2 cells with selected DsiRNA swarms. Cells were mock transfected (non-infected and no-DsiRNA controls) or pre-transfected with control (ctrl) DsiRNA, chimeric (chim) DsiRNA or selected SARS-CoV-2 specific DsiRNAs for 21 h. Cells were subsequently infected with Fin-3 SARS-CoV-2 at a MOI of 0.1 or 0.01. **(A)** Total RNAs were isolated from the cell culture supernatant samples, and the vRNA levels were analyzed by RT-qPCR. The results are the mean values ± SEM of three independent experiments. Statistical significance was determined against results of samples of DsiRNA non-transfected cells (boxed bars). *P < 0.05. **(B)** The infectious virus titers were analyzed from the supernatants at different time points (1, 24, and 48 h p.i.) by endpoint dilution assay in VE6-T2 cells. Results are shown as TCID_50_/ml and represent the means ± SEM from three independent experiments. Statistical significance was determined against results of samples of DsiRNA non-transfected cells (boxed bars). *P < 0.05.

Next, we analyzed whether the pre-transfection of SARS-CoV-2 DsiRNAs could block the secretion of infectious SARS-CoV-2. For a more comprehensive analysis, vRNA quantity and infectious particle titers were analyzed from the supernatant samples of SARS-CoV-2 Fin-3 infected VE6-T2 cells collected at 1, 24, or 48 h p.i. with and without pre-transfection of SARS-CoV-2 DsiRNAs. The amount of vRNA in Fin-3 infected cell culture supernatant samples (both at MOI 0.1 and 0.01) without pre-transfection of SARS-CoV-2 DsiRNA swarms increased at 24 h, compared with the 1 h p.i. incoming virus sample, reaching approximately 10^9^ copies/ml at 48 h p.i. (Figure 4A). The copy number of vRNA in the supernatant of Fin-3 infected cells with pre-transfection of control DsiRNA was at a similar level as the one without pre-transfection of DsiRNAs. Pre-transfection of cells with the chimeric or the four SARS-CoV-2 gene/UTR-specific DsiRNA swarms largely reduced the copy number of vRNA in the supernatant of Fin-3 infected cells. Evident reduction of vRNA levels was observed at 24 h p.i., reaching over a 2-log significant reduction at 48 h p.i. (Figure 4A). The endpoint dilution assay, quantitating secreted infectious virions, confirmed the vRNA copy number results (Figure 4B). The virus titer in the supernatant of cells with the pre-transfection of the chimeric and the four SARS-CoV-2 gene/UTR-specific DsiRNAs at 48 h p.i. with high MOI value of 0.1 was reduced approximately 2-logs compared to the supernatant from Fin-3 infected cells without DsiRNA pre-transfection, and was reduced up to 3 logs when cells were infected at a low MOI value of 0.01. Pre-transfection of control DsiRNAs did not inhibit the secretion of infectious virions.

Previously, we have shown that DsiRNAs against influenza A virus (IAV) do not induce IFN responses in human primary cells (Jiang et al., 2019). In the current study, we also studied the off-target effects induced by DsiRNAs in VE6-T2 cells. Since type I IFN signaling pathway is lacking in VE6-T2 cells (Chew et al., 2009; Prescott et al., 2010; Lokugamage et al., 2020), we tested type III *IFN-*λ*1* gene expression induced by SARS-CoV-2 specific DsiRNAs. VE6-T2 cells were transfected with control DsiRNAs as well as SARS-CoV-2 DsiRNAs, and total cellular RNAs were extracted 21 h p.t.. 5′triphosphate-containing 88bp dsRNA which can induce very pronounced IFN responses in primary human immune cells (Jiang et al., 2011) was transfected as a positive control. RT-qPCR results showed that the *IFN-*λ*1* mRNA expression was not markedly elevated by transfection with any of the DsiRNAs, whereas 88bp dsRNA significantly induced high level of *IFN-*λ*1* mRNA expression (Supplementary Figure S2). A cell viability assay also showed that transfection of DsiRNAs failed to induce any significant cytotoxicity in SARS-CoV-2 non-infected and infected VE6-T2 cells (Supplementary Figure S3).

### 3.5 Comparison of Inhibition efficacy of SARS-CoV-2 DsiRNAs in VE6-T2 cells when DsiRNAs were administered prior to and after the infection of SARS-CoV-2

**Figure 5:**
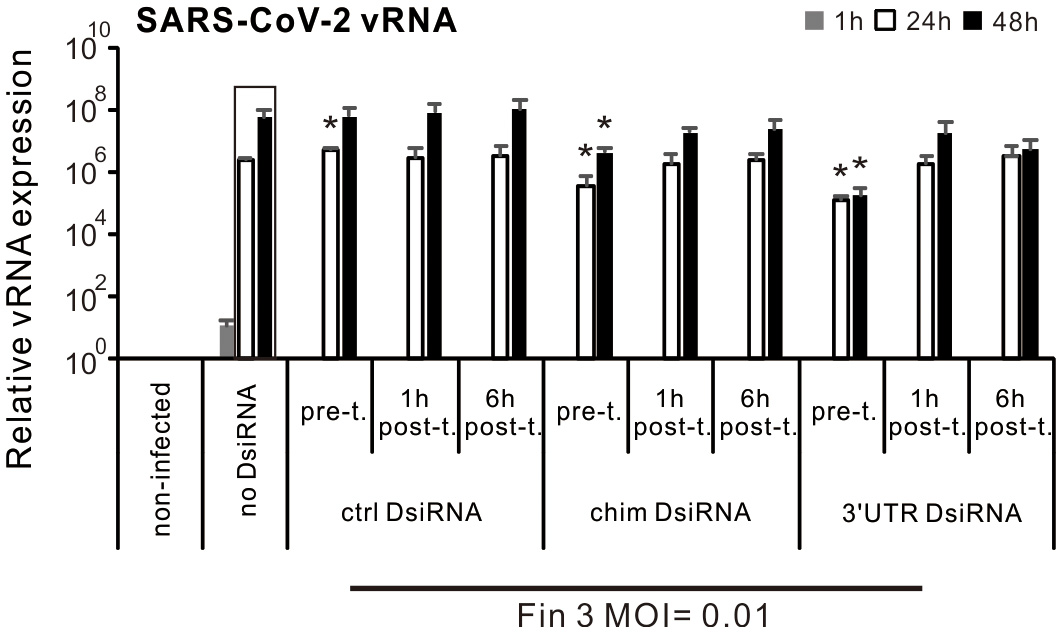
Comparison of Inhibition efficacy of SARS-CoV-2 DsiRNAs in VE6-T2 cells with different DsiRNA delivery time points. Cells were mock transfected (non-infected and no-DsiRNA controls) or pre-transfected with control (ctrl) DsiRNA, chimeric (chim) DsiRNA or 3′UTR SARS-CoV-2 specific DsiRNAs 21 h before (pre-t.), or 1 h and 6 h after the infection (post-t.) with Fin-3 SARS-CoV-2 at a MOI 0.01. Cells were then collected at 1, 24, and 48 h p.i., and SARS-CoV-2 vRNA expression was analyzed from isolated total cellular RNA samples by RT-qPCR. The SARS-CoV-2 E vRNA Ct values were normalized against human 18S rRNA, and relative SARS-CoV-2 vRNA levels were calculated by the ΔΔCT method using untreated cellular RNA as a calibrator. The means (±SEM) of three parallel analyses are shown. Data is a representative of three individual experiments. Statistical significance was determined against results of samples of DsiRNA non-transfected cells (boxed bars). *P < 0.05.

Our previous IAV study showed that IAV DsiRNAs can inhibit IAV replication in human moDCs only when administered prior to infection (Jiang et al., 2019). To also study the optimal time of SARS-CoV-2 DsiRNA delivery for antiviral effects, VE6-T2 cells were transfected with SARS-CoV-2 specific DsiRNAs 21 h before, 1 h or 6 h after the infection by Fin-3 (MOI of 0.01). Cells were collected and total RNAs were isolated from the infected cells at 1, 24, and 48 h p.i., and vRNA expression of the SARS-CoV-2 was analyzed by gene-specific RT-qPCR. Pre-transfection with the chimeric and 3′UTR-specific DsiRNA swarms led to ca. 1–2 log significant reduction in the expression level of vRNA at 24 h and 48 h p.i. compared to the non-transfected Fin-3 infected control (no DsiRNA), while transfection with all studied DsiRNAs after SARS-CoV-2 infection (both 1 h and 6 h) failed to significantly inhibit viral RNA expression of Fin-3 in VE6-T2 cells (Figure 5).

### 3.6 Inhibition of the replication and productive infection of SARS-CoV-2 Omicron variants in VE6-T2 cells by SARS-CoV-2 specific chimeric, 3′UTR, NSP1, and NSP2 DsiRNA swarms

**Figure 6:**
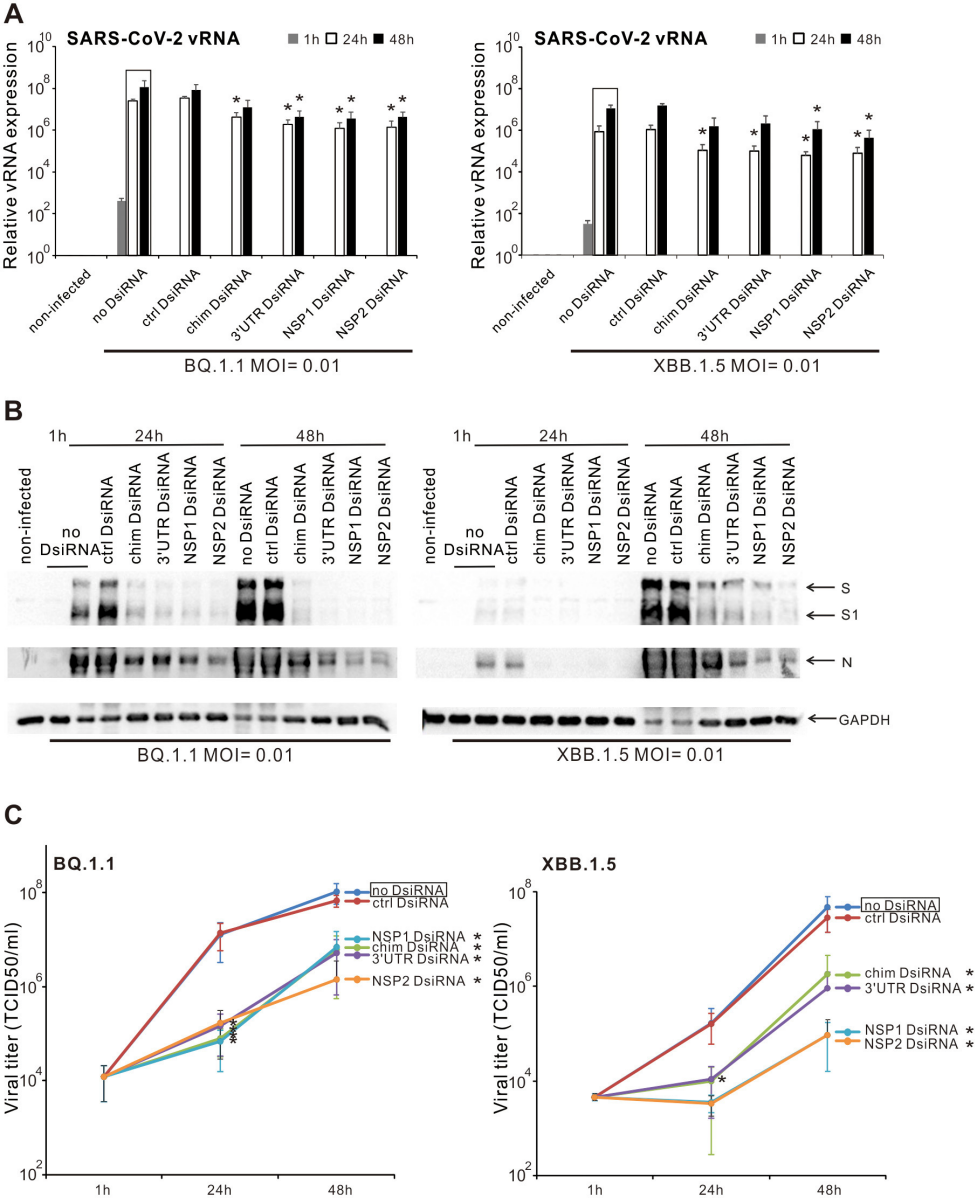
Inhibition of the replication and production of Omicron variants by DsiRNA swarms in VE6-T2 cells. Cells were mock transfected (non-infected and no-DsiRNA controls) or pre-transfected with control (ctrl) DsiRNA, chimeric (chim) DsiRNA or selected SARS-CoV-2 specific DsiRNAs for 21 h. Cells were subsequently infected with BQ.1.1 and XBB.1.5 variants of SARS-CoV-2 at a MOI value of 0.01. SARS-CoV-2 vRNA expression **(A)**, the expression of viral S and NP proteins **(B)**, and infectious viral titers from the culture supernatants **(C)** were analyzed. The means (±SEM) of three parallel analyses are shown. Data is representative of three individual experiments. Statistical significance was determined against results of samples of DsiRNA non-transfected cells (boxed bars). *P < 0.05.

Next, we investigated whether the chimeric and SARS-CoV-2 specific DsiRNA swarms also inhibit the replication of Omicron variants of SARS-CoV-2. The Omicron variants and subvariants are still predominant viruses with a higher number of mutations (especially in the spike gene) and a stronger transmission ability. VE6-T2 cells were pre-transfected with control, chimeric, 3′UTR-, NSP1-, and NSP2-specific SARS-CoV-2 DsiRNA swarms, followed by infection with BQ.1.1 or XBB.1.5 variants of SARS-CoV-2 at 21 h p.t. at MOI 0.01. Total cellular RNAs, cellular proteins, and supernatant samples were collected at 1, 24, and 48 h p.i.. RT-qPCR, Western blotting and end-point dilution assay were carried out to measure the inhibitory efficacy of SARS-CoV-2 DsiRNA swarms against Omicron infection in VE6-T2 cells at vRNA, viral protein and infectious virus titer level, respectively. The vRNA expression of both BQ.1.1 and XBB.1.5 variants was reduced approximately 1 log by pre-transfection of the cells with the chimeric DsiRNA swarm or the 3′UTR-, NSP1-, and NSP2-specific DsiRNA swarms (Figure 6A). Pre-transfection of BQ.1.1 infected cells with the chimeric DsiRNA swarm or the 3′UTR-, NSP1-, and NSP2-specific DsiRNA swarms almost completely blocked the S protein expression and its cleavage to S1 at both 24 h and 48 h p.i., and greatly reduced the expression of N protein, especially at 48 h p.i.. In XBB.1.5 infected cells, the pre-transfection of SARS-CoV-2 DsiRNA swarms also completely inhibited S and N protein expression and the S cleavage to S1 at 24 h p.i., while only largely reduced the S protein cleavage and the expression of S and N protein at 48 h p.i. (Figure 6B). The end-point dilution assay also showed that the pre-transfection of both chimeric and 3′UTR, NSP1, and NSP2 specific DsiRNA swarms led to approximately 1-3 log reduction in the secretion of infectious BQ.1.1 or XBB.1.5 viruses at 48 h p.i., indicating a strong inhibitory efficacy of these DsiRNA swarms against the replication of Omicron variants in VE6-T2 cells (Figure 6C).

## 4 Discussion

Previously, we have introduced a novel enzymatic approach to synthesize DsiRNA swarms containing a number of different target-specific DsiRNAs instead of single chemically synthetized canonical siRNAs, and have validated the antiviral efficacy of such swarms against the infection of various strains of influenza A viruses in different types of cells (Jiang et al., 2019). In the present study, we use the same approach to synthesize a chimeric DsiRNA swarm targeting multiple genes of SARS-CoV-2, as well as specific DsiRNA swarms targeting single genes/UTRs of SARS-CoV-2, including 15 NSPs, 4 structural proteins, as well as 5′- and 3′-UTRs. We identified and validated the antiviral efficacy of the novel DsiRNA swarms against the infection of SARS-CoV-2 in VE6-T2 cells. Moreover, we demonstrated that these novel DsiRNA swarms effectively inhibit the replication of different SARS-CoV-variants in VE6-T2 cells, including early strain Fin-3 and recent Omicron variants, BQ.1.1 and XBB.1.5.

Currently, siRNAs under development for SARS-CoV-2 therapeutics are mainly targeting the 5′ leader sequence of the SARS-CoV-2 genome or genomic regions encoding the viral RdRp complex, helicase, or structural proteins since these siRNAs have shown high potential to inhibit SARS-CoV-2 infection (Idris et al., 2021; Khaitov et al., 2021; Shawan et al., 2021; Tolksdorf et al., 2021; Wu and Luo, 2021; Ambike et al., 2022). In this study, we systematically screened the antiviral efficacy of DsiRNA swarms targeting 5′- and 3′-UTRs as well as all the protein coding regions of SARS-CoV-2, except NSP11 which encodes a small, 13 amino acids long peptide. Like in previous studies, here we show that SARS-CoV-2 Fin-3 infection in VE6-T2 cells is suppressed by DsiRNA swarms targeting 5′-UTR, NSP7, NSP8, NSP13, and especially by NSP12 DsiRNA swarm. As presently known, 5′-UTR is essential for the initiation of SARS-CoV-2 protein translation (Miao et al., 2021; Condé et al., 2022) and for bypassing the host protein translation blockage mediated by NSP1 (Vora et al., 2022). RdRp complex, composed of NSP7, NSP8, and NSP12, is responsible for the replication and transcription of the viral RNAs (Hillen et al., 2020). NSP12 possesses a nucleotidylation activity to form the 5′ cap structure (Yan et al., 2021), and NSP13 has NTPase and RNA helicase activities supporting unwinding of viral RNA duplexes and supplying energy for viral RNA elongation (Shu et al., 2020). SiRNAs targeting and degrading these essential viral genes and non-coding regulatory elements can largely reduce or block the transcription and replication of viral RNAs, and therefore restrict the replication of SARS-CoV-2.

Besides the above-mentioned RNAi targets in the SARS-CoV-2 genome identified in previous studies (Idris et al., 2021; Khaitov et al., 2021; Shawan et al., 2021; Tolksdorf et al., 2021; Wu and Luo, 2021; Ambike et al., 2022) and confirmed by us (Figure 2), we identified and substantiated the inhibitory efficacy of DsiRNA swarms targeting three other regions in the SARS-CoV-2 genome: 3′-UTR, NSP1, and NSP2. Pre-transfection of any of these three DsiRNA swarms into cells greatly inhibited the replication of SARS-CoV-2 vRNAs and completely blocked the expression of viral S and N proteins at 48 h p.i. in SARS-CoV-2 infected cells at a low MOI value (MOI 0.01) (Figure 3). Moreover, these DsiRNA swarms showed a pronounced antiviral efficacy by inhibiting the production and secretion of progeny SARS-CoV-2 viruses (Figure 4). The newly identified target region 3′-UTR is a highly conserved region in the SARS-CoV-2 genome and it contains several essential RNA elements which may contribute to the synthesis and translation of viral RNAs (Cao et al., 2021). While the therapeutic potential of this region as an RNAi target has been predicted previously (Park and Moon, 2022), our study provided solid experimental evidence for its functionality. The identified RNAi target gene NSP1 encodes a viral leader protein, which is mainly responsible for translational shut down of host proteins by promoting the degradation of host mRNAs (Kamitani et al., 2009; Thoms et al., 2020; Vankadari et al., 2020). Moreover, the N-terminal domain of NSP1 binds to a specific stem-loop 1 region in the 5′-UTR of SARS-CoV-2 RNAs, releasing the interaction between the C-terminal domain of NSP1 and the eukaryotic 40S small ribosomal subunit and leading to the recruitment of endonuclease by NSP1, thus allowing SARS-CoV-2 RNAs to avoid translational suppression and endonucleolytic RNA cleavage by itself (Min et al., 2020; Vankadari et al., 2020; Vora et al., 2022). The pre-transfection of DsiRNA swarm targeting *NSP1* gene may reduce NSP1 protein levels and block the binding of NSP1 to host ribosomal subunits and to stem-loop 1 region of SARS-CoV-2 RNAs, leading to an imbalance in the control of RNA degradation and protein translation between SARS-CoV-2 and the host cell RNAs, and therefore inhibiting the replication of the virus. The function of the identified target region NSP2 has not been fully identified. It has been shown that NSP2 can non-specifically bind to nucleic acids, linking the transcription to the initiation of translation (Ma et al., 2021; Verba et al., 2021). Moreover, mutations in NSP2 are highly related to increased transmissibility and pathogenicity of SARS-CoV-2 variants (Flores-Alanis et al., 2021; Patro et al., 2021; Wang et al., 2021). Our results show that pre-transfection of NSP2 DsiRNA swarm effectively inhibits the expression of SARS-CoV-2 vRNA and proteins, including those of Omicron variants. NSP2 DsiRNA swarm also inhibited the progeny virus production and secretion, suggesting its role as a promising novel therapeutic candidate for the inhibition of SARS-CoV-2 replication and the treatment of COVID-19.

Unexpectedly, pre-transfection of DsiRNA swarm targeting NSP10 and N failed to inhibit the replication of SARS-CoV-2. NSP10 can bind to both NSP14 and NSP16 and act as a scaffold and a stimulatory protein for these two enzymes (Krafcikova et al., 2020; Wang et al., 2023). The N protein, however, is a structural protein that binds to viral RNA, packages RNA genome into ribonucleoprotein (RNP) complex and maintains the stability of RNP complex (Kang et al., 2020; Zeng et al., 2020; Wu W. et al., 2023). N protein plays an essential role in viral RNA replication and transcription. A failure in inhibition of replication of SARS-CoV-2 by NSP10 and N DsiRNA swarms may be due to a minor function role of NSP10 during the transcription and replication of SARS-CoV-2, and due to the high abundance of expressed N transcripts and N proteins during the replication of SARS-CoV-2 (Kim et al., 2020; Wu J.-L. et al., 2023), respectively. This might also be the reason why the chimeric DsiRNA swarm, containing targets of N gene, shows a weaker antiviral efficacy against the infection of SARS-CoV-2 as compared to DsiRNA swarms targeting other SARS-CoV-2 genes or UTRs.

The emergence of Omicron variants starting in autumn 2021 quickly led to a peak in global COVID-19 morbidity with 1 to 4 million reported daily cases in the winter of 2021 to 2022. Comparing with early ancestral viruses, Omicron variants contained more than 40 amino acid substitutions in their S protein (Viana et al., 2022), enabling evasion from infection- or vaccine-induced immunity in the population. This phenomenon led Omicron variants to become the dominant viruses globally. The appearance of Omicron variants represented a new stage in the COVID-19 pandemic, as they possessed a much higher capacity to re-infect people than any of the previous SARS-CoV-2 variants (Pulliam et al., 2022) and Omicron sub-variants are still spreading in the continuing COVID-19 epidemics. In order to test the antiviral efficacy of our SARS-CoV-2 DsiRNA swarms against Omicron variants, the chosen DsiRNA swarms targeting 3′-UTR, NSP1, and NSP2, as well as chimeric SARS-CoV-2 swarm, were pre-transfected into cells, followed by an infection with two prevalent Omicron variants (BQ.1.1 and XBB.1.5) identified in 2022 and 2023, respectively. The results showed that our chimeric as well as 3′-UTR, NSP1, and NSP2 specific SARS-CoV-2 DsiRNA swarms efficiently inhibit the expression of vRNA and proteins, and importantly, also inhibit the production of progeny viruses in the supernatants of Omicron-infected cells. This suggests a broad antiviral efficacy of our SARS-CoV-2 DsiRNA swarms against the infection of different variants of SARS-CoV-2.

VE6 cells are an ideal cell model to study viruses due to the highly permissive nature to various viruses, including SARS-CoV-2 (Chew et al., 2009). The signaling of type I IFN pathway in VE6 cells is defective (Chew et al., 2009; Prescott et al., 2010; Lokugamage et al., 2020), and thus the type I IFN signaling should be deficient in VE6-T2 cells as well. Moreover, it has been reported that weak IRF3-dependent responses to different stimuli of ligands (regulating both type I and type III gene expressions) result in overall very weak innate immune responses in Vero cells (Chew et al., 2009). Therefore, the inhibition of the infection of SARS-CoV-2 variants in VE6-T2 cells is very likely due to the virus-specific gene silencing induced by our SARS-CoV-2 DsiRNA swarms instead of DsiRNA-induced IFN responses. As the products digested by Dicer, DsiRNAs are 25-27-mer long which is slightly longer than traditional siRNA with the size of 21-23-mer. Therefore, the longer siRNAs may have a higher risk of off-target effects. In the current study, we analyzed the potent off-target effects induced by our SARS-CoV-2 DsiRNAs and the results showed the IFN responses as well as cytotoxicity induced by our DsiRNAs were very moderate in our model cell system (Supplementary Figures S2, S3). A failure to induce mRNA expression of IFN-λ1 by DsiRNA further demonstrated that the inhibition of SARS-CoV-2 in VE6-T2 cells is due to gene silencing induced by DsiRNAs. Moreover, a previous study also showed that DsiRNA is up to 10 or 100-fold more potent in silencing the target gene than the traditional 21-mer siRNAs (Song et al., 2022). In addition, our DsiRNA swarms contain tens or hundreds of DsiRNAs targeting different regions of genes, which can minimize the off-target effects. Therefore, all the data has demonstrated that SARS-CoV-2 DsiRNA swarm can efficiently inhibit the replication of the virus with a very low risk of inducing off-target effects in VE6-T2 cells.

The delivery of siRNAs or DsiRNAs into the cells is essential for antiviral therapy. Previously, we have demonstrated the high transfection efficiency of TransIT-X2 for siRNA delivery (Jiang et al., 2019). In the current study, we used the same siRNA delivery method and observed that the cell viability of VE6-T2 with the transfection of SARS-CoV-2 DsiRNA swarms did not change even at day 3 p.t. (Supplementary Figure S3), suggesting that the siRNA delivery system used in the study was safe and efficient. As demonstrated here, prophylactic treatment with SARS-CoV-2 DsiRNA swarms efficiently inhibits the SARS-CoV-2 replication in VE6-T2 cells. However, transfection of DsiRNA swarms after the infection lost their inhibitory efficacy against the replication of the virus. This result was consistent with our previous result in an IAV study (Jiang et al., 2019). As mentioned in the previous study, our data can not be simply interpreted as that pre-infection delivery of DsiRNA is an essential prerequisite for clinical efficacy, while it is possible that during early phases of SARS-CoV-2 infection most of the epithelial cells in the respiratory tissues of a patient are still uninfected, and thus there may be a window phase for DsiRNAs delivery to prevent the further spread of the infection. However, the antiviral efficacy of therapeutic treatment with DsiRNA swarms in SARS-CoV-2 infection needs to be verified in further *in vivo* experiments. Previously, most siRNA therapeutic approaches against SARS-CoV-2 have relied on single-site chemically synthesized siRNA molecules. Compared to these, our approach of DsiRNA swarm production has a better feasibility for industrial mass production and clinical use due to lower expenses as well as a lower risk of off-target side effects.

## 5 Conclusion

Our previous study showed that by utilizing bacteriophage T7 DNA-dependent RNA polymerase, bacteriophage ϕ6 RNA-dependent RNA polymerases and *Giardia intestinalis* Dicer, we produced a broad-spectrum influenza A virus-specific DsiRNA swarm *in vitro* that was efficient in inhibiting the replication of different influenza virus strains in various human cells (Jiang et al., 2019). In the current study, we produced a set of DsiRNA swarms targeting different non-coding regions or genes of SARS-CoV2 as well as a chimeric DsiRNA swarm targeting multiple genes of SARS-CoV-2 genome by using a similar *in vitro* DsiRNA producing system. We screened the antiviral efficacy of all the produced DsiRNA swarms against SARS-CoV-2 infection in VE6-T2 cells. Moreover, we validated the antiviral efficacy of chosen DsiRNA swarms, including the chimeric DsiRNA swarm, and DsiRNA swarms targeting 3′-UTR, NSP1, NSP2, and NSP12, by measuring the changes in vRNA transcription and viral protein expression, and in the production and secretion of progeny SARS-CoV-2 viruses after DsiRNA swarm treatment. We also demonstrated an efficient antiviral activity of our SARS-CoV-2 DsiRNA swarms, targeting 3′-UTR, NSP1, and NSP2 of SARS-CoV-2, on emerged Omicron variants. Our results suggest that these RNAi targets are ideal for further design of siRNA therapy against COVID-19. This study provides solid evidence for the feasibility of a new siRNA strategy for the prevention and treatment of SARS-CoV-2 infection. It will also help us develop new prevention strategies and therapeutic interventions for tackling both previously known and yet unidentified threats from the continuously evolving SARS-CoV-2 variants.

## Data Availability

The original contributions presented in the study are included in the article/Supplementary material, further inquiries can be directed to the corresponding authors.
