# Supplementary figures and images for: Virus-specific Dicer-substrate siRNA swarms inhibit SARS-CoV-2 infection in TMPRSS2-expressing Vero E6 cells
Source: Front Microbiol. 2024 Nov 14;15:1432349. doi: 10.3389/fmicb.2024.1432349 (PMC11602746; doi:10.3389/fmicb.2024.1432349)

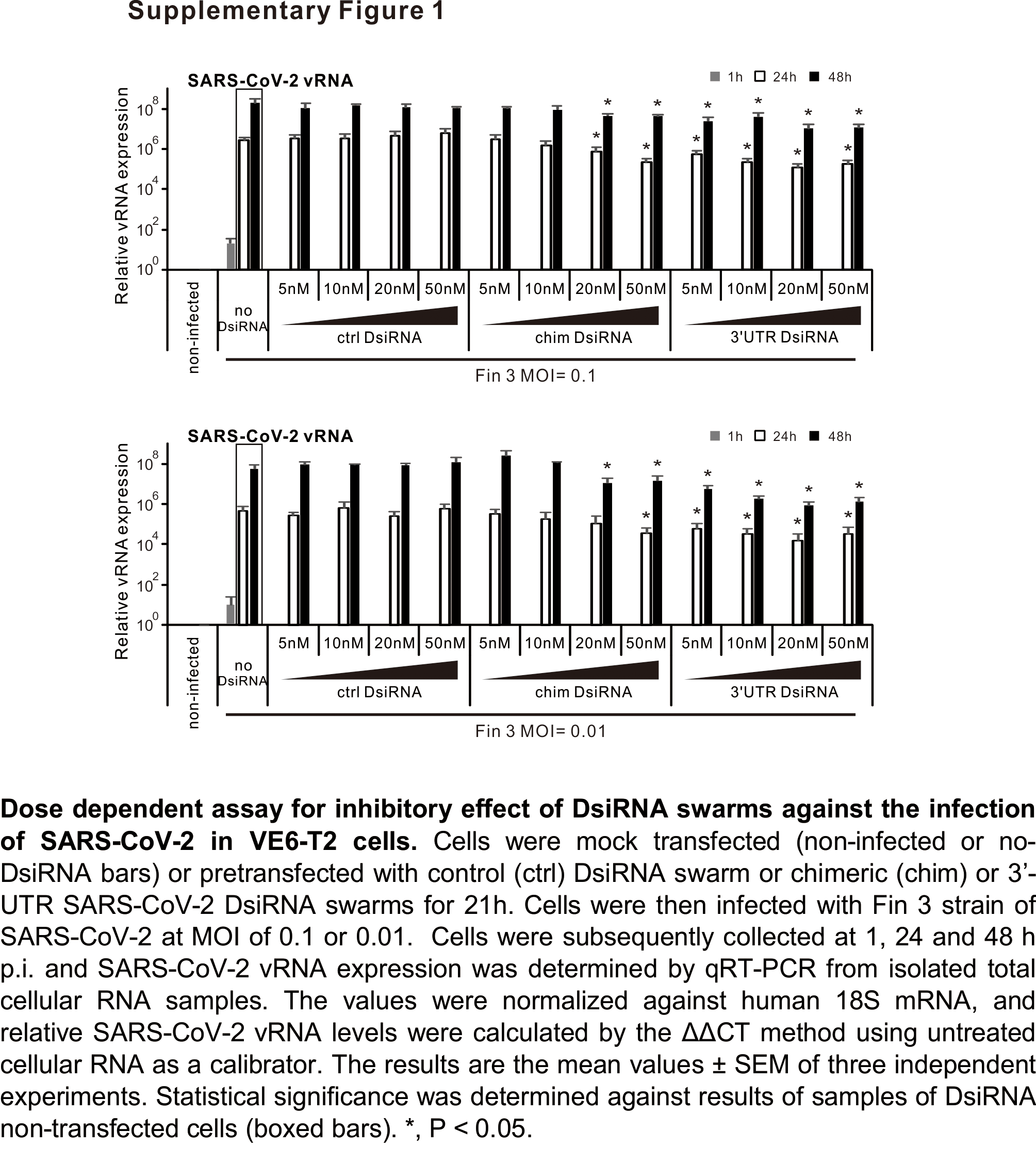

Supplement: Supplementary file 1 [file Image_1.tif]

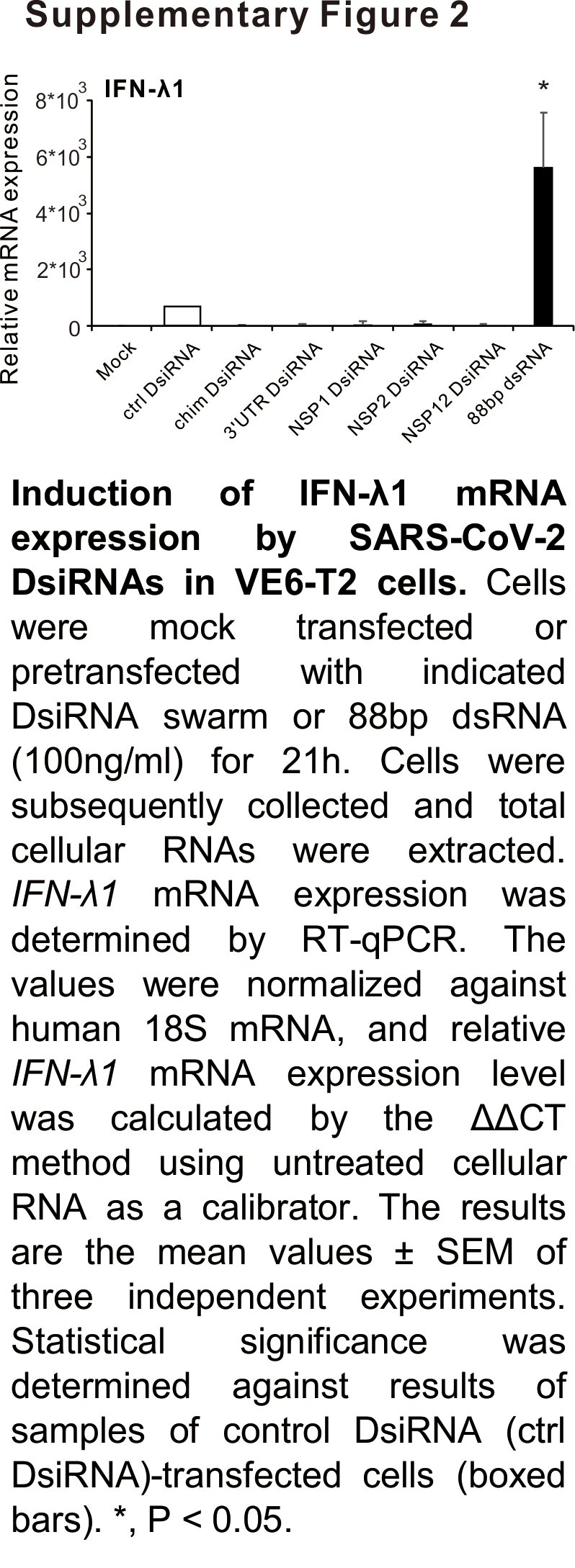

Supplement: Supplementary file 2 [file Image_2.tif]

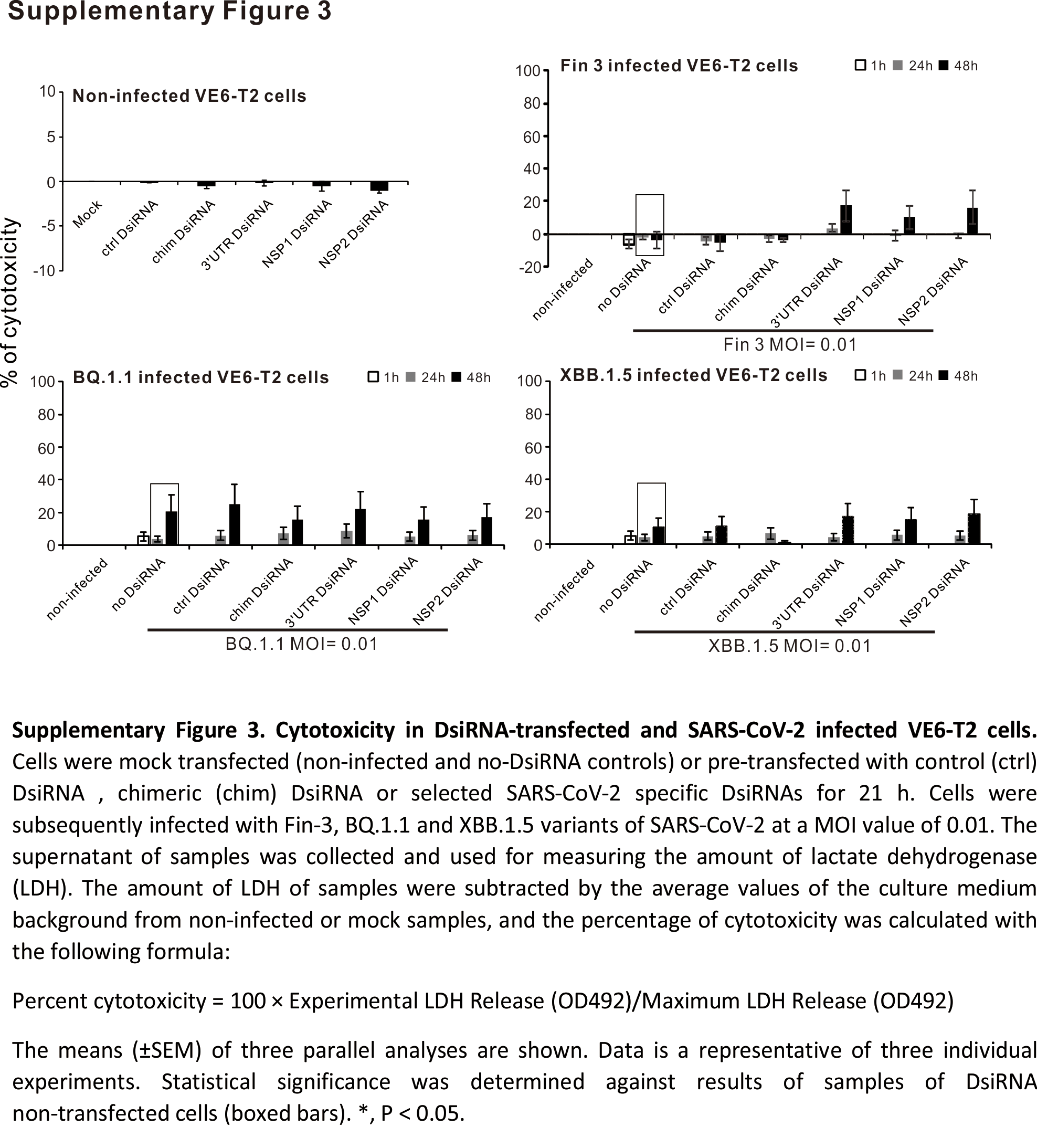

Supplement: Supplementary file 3 [file Image_3.tif]
